# Autoimmune-Like Hepatitis Following Hepatic Graft-Versus-Host Disease in an Allogenic Hematopoietic Cell Transplant Recipient

**DOI:** 10.1155/crhe/8411674

**Published:** 2025-08-13

**Authors:** Harish Gopalakrishna, David E. Kleiner, Jennifer A. Kanakry, Marc G. Ghany

**Affiliations:** ^1^Department of Liver Diseases, National Institute of Diabetes and Digestive and Kidney Diseases, National Institutes of Health, Bethesda, Maryland, USA; ^2^Department of Pathology, National Cancer Institute, National Institutes of Health, Bethesda, Maryland, USA; ^3^Department of Immuno-Oncology, Center for Cancer Research, National Cancer Institute, National Institutes of Health, Bethesda, Maryland, USA

**Keywords:** autoimmune-like hepatitis, graft-versus-host disease, GVHD, hematopoietic cell transplant

## Abstract

One of the major complications following hematopoietic cell transplantation (HCT) is the occurrence of graft-versus-host disease (GVHD). The liver is a target organ in both acute and chronic GVHD. Histologically, there are two distinct forms of hepatic involvement by GVHD, namely cholestatic (classical) and hepatic. Autoimmune-like hepatitis has been reported as a late complication of HCT with some considering it to be a variant of hepatic GVHD. However, there are no reports of hepatic form of GVHD and autoimmune-like hepatitis in the same patient post-HCT. Herein, we report a patient who initially developed hepatic GVHD followed by autoimmune-like hepatitis.

## 1. Introduction

Graft-versus-host disease (GVHD), a frequent and serious complication following allogeneic hematopoietic cell transplantation (HCT), commonly involves the skin, gastrointestinal tract (GI), and liver [1]. GVHD is a T-cell-mediated process whereby the transplanted T lymphocytes target host antigens, resulting in tissue injury [2]. In the liver, donor T-cells target the endothelium and cholangiocytes, leading to endothelialitis, pericholangitis, and apoptotic bile duct destruction [3]. Clinically, liver GVHD can present in two patterns, cholestatic (classical) or hepatic [4]. The classical pattern is more common, presenting with elevated alkaline phosphatase and total bilirubin, and is associated with bile duct injury and cholestasis with minimal to no lobular inflammation [5]. In contrast, the hepatic pattern is characterized by elevated transaminases and prominent lobular inflammation with minimal bile duct injury [6]. In addition, an autoimmune-like hepatitis (AIH) has been described post-HCT [7–9]. This is histologically different from typical liver GVHD, characterized by interface hepatitis with lymphoplasmacytic portal and periportal infiltration that some consider to be a variant of hepatic GVHD [10]. However, there are no reports of both entities, hepatic GVHD and AIH, occurring in the same patient.

## 2. Case Report

A 38-year-old Honduran male with peripheral T-cell lymphoma (PTCL) received 10/10 HLA-matched, alloimmune ABO compatible, peripheral HCT from his sister. His conditioning regimen consisted of equine antithymocyte globulin (3 gm × 2 doses), pentostatin (7.2 mg × 2 doses), hyperfractionated cyclophosphamide (400 mg daily × 8 days), and pharmacokinetically dosed busulfan for 2 days. GVHD prophylaxis included cyclophosphamide (50 mg/kg/day on Days 3 and 4 post-HCT), sirolimus from Day 5 through 60 post-HCT (goal trough 5–12 ng/mL), and mycophenolate mofetil (1 gm thrice daily) from Days 5 through 25 post-HCT. He was engrafted 2 weeks later with 100% donor cells in myeloid and T-cell compartments and did not develop acute GVHD.

**Figure 1:**
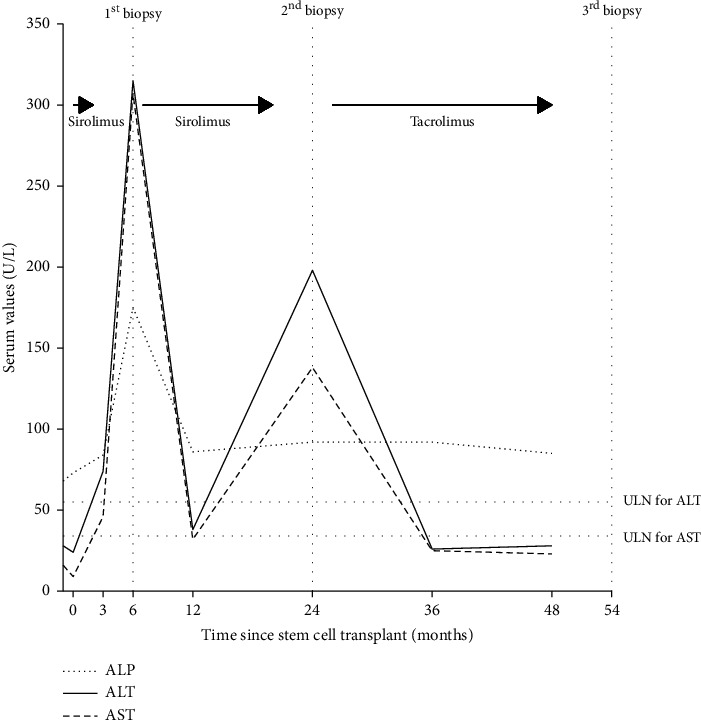
Trend of liver-associated enzymes in relation to immunosuppressant medications and the timing of liver biopsies. Abbreviations: ALT: alanine aminotransferase, AST: aspartate aminotransferase, ALP: alkaline phosphatase, U/L: units per liter, ULN: upper limit of normal.

On Day 99 post-HCT, he presented with diagnostic findings of chronic GVHD involving the mouth and genitals. On Day 119 post-HCT, he had > 3-fold elevation in transaminases (Figure 1). There were no identifiable risk factors for liver disease aside from recurrent episodes of binge alcohol consumption.

**Figure 2:**
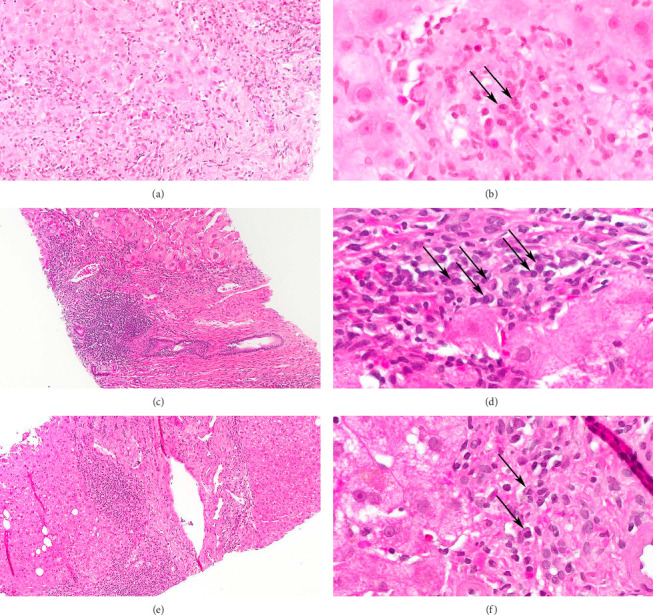
Evolution of hepatitis. The initial biopsy showed hepatitis with lobular, portal, and periportal inflammation with eosinophils (a) and mild duct injury (not shown). Examination at high magnification identified rare plasma cells (b). The second biopsy showed typical features of autoimmune hepatitis, including dense portal inflammation with interface hepatitis (c) and clusters of plasma cells (d). The most recent biopsy showed a reduced amount of portal inflammation and interface hepatitis (e) with only rare plasma cells (f). H&E stains, magnification A, 200x; C, E; 100x, B, D, F 600x.

On examination, there were no features of chronic liver disease; there was evidence of an erythematous maculopapular rash involving 5% body surface area on his bilateral upper extremities. Laboratory studies revealed normal complete blood count, creatinine, iron panel, and α-fetoprotein. Evaluation for liver diseases including viral markers (hepatitis A, B, and C), genetic, and autoimmune serological panels including anti-liver kidney microsomal 1 antibody (Table 1) were negative. Ultrasound of the liver was normal. Percutaneous liver biopsy revealed mild to moderate portal and lobular inflammation with eosinophilic infiltrates and only rare plasma cells; there was no cholestasis, but bile ducts showed injury with reactive changes. Findings were consistent with the hepatic variant of GVHD (Figures 2(a) and 2(b)). Initial treatment with prednisone 0.5 mg/kg/day for 5 days, along with rituximab 4 doses weekly, led to improvement in transaminases, but they subsequently rose again. Prednisone was restarted at 0.5 mg/kg/day for 4 days, followed by a slow taper, along with sirolimus 2 mg daily as a steroid-sparing agent. He developed ocular GVHD on Day 148 and lung GVHD on Day 176 post-HCT. Eventual remission of GVHD in all organs allowed sirolimus to be stopped at 1.5 years post-HCT.

At 1.7 years post-HCT, he had recurrent elevation of liver enzymes (Figure 1). GVHD remained quiescent in other organs despite being off of systemic immunosuppression. He was not on any hepatotoxic drugs, and repeat viral hepatitis workup was negative (Table 1). A repeat liver biopsy demonstrated a moderate chronic portal inflammatory infiltrate associated with moderate interface hepatitis, clusters of plasma cells, as well as scattered eosinophils with the absence of bile duct injury. These histological findings were consistent with AIH (Figures 2(c) and 2(d)). The patient's score based on the modified criteria of the International Autoimmune Hepatitis Group was 14, suggestive of probable AIH. Prednisone 40 mg daily was started as induction therapy with subsequent improvement in liver enzymes. Two months later, tacrolimus 1.5 mg twice daily was added for maintenance therapy, and prednisone was slowly tapered off. Nine months later, the tacrolimus dose was tapered to 1 mg twice daily resulting in mild elevation of liver enzymes, which later normalized. He remained on maintenance tacrolimus 1 mg twice daily for 2 years with normal liver enzymes. While on immunosuppressant therapy, the patient experienced recurrent viral infections requiring hospitalization. A repeat liver biopsy was performed to assess whether immunosuppression could be stopped. The biopsy showed persistent mildly active hepatitis and portal fibrotic expansion despite normal liver enzymes (Figures 2(e) and 2(f)). Given these histological findings, it was decided to continue on tacrolimus at the same dose.

## 3. Discussion

About 4%–30% of liver GVHD cases manifest as the hepatic variant [11, 12]. Risk factors include use of an unrelated donor, HLA mismatch, sex disparity, peripheral HCT, and donor lymphocyte infusion [13, 14]. In this case, the only risk factors were donor-recipient sex disparity and peripheral blood stem cell graft. The hepatic variant has a similar but slower response to treatment as compared to the classical form [6]. Our patient responded well and remained on treatment for over a year.

Despite stable GVHD involvement of other organs, the patient experienced recurrent elevation in transaminase levels. The pattern of histological injury, absence of bile duct involvement, and clinical response to steroids supported a diagnosis of AIH-like hepatitis. There is some consideration that AIH-like hepatitis occurring after HCT might be a variant of hepatic GVHD [15]. However, there have been no prior reports of its occurrence following an earlier episode of biopsy-proven hepatic GVHD. Both hepatic GVHD and AIH-like hepatitis can occur after immunosuppressant tapering [7, 8], but they differ in histology and timing of occurrence. The hepatic form of liver GVHD typically presents 4–5 months post-HCT, whereas AIH-like hepatitis usually occurs after a median of 1 year post-HCT [10].

Both autoantibody positive and negative AIH-like hepatitis have been reported [16, 17]. Although our patient had neither autoantibodies nor hypergammaglobulinemia, the IgG levels almost doubled from his baseline levels. It is also unclear whether the guidelines defined by the modified criteria of the International Autoimmune Hepatitis Group to define AIH are applicable in this scenario [18], since the underlying pathophysiology remains poorly understood. Proposed mechanisms include immune dysfunction with reconstitution post-HCT or due to transfer of autoantibodies from the donor [19]. Given the rarity of this condition, the appropriate treatment regimen, duration of treatment, and markers of disease activity are not well understood. Following steroid induction, tacrolimus has been successful in maintaining clinical but not histological remission [7, 10]. In this case, the decision to use calcineurin inhibitors was based on previous reports supporting their effectiveness [10]. However, alternative treatments such as azathioprine have also been reported to yield successful outcomes [8].

## 4. Conclusion

We report a unique case with long-term histological demonstration of AIH-like hepatitis occurring after hepatic GVHD in a patient post-HCT. AIH-like hepatitis is currently considered a variant of hepatic GVHD [10], but our case suggests the possibility of AIH-like hepatitis being a continuum of hepatic GVHD. Alternatively, it is also possible that both represent separate de novo processes. Further studies with long-term follow-up of patients who develop hepatic GVHD are necessary to understand the natural history of hepatic GVHD.

## Figures and Tables

**Table 1:** Laboratory parameters in relation to hematopoietic stem cell transplant.

**Laboratory parameters**	**Pre-HCT**	**HCT**	**Post-HCT**					
	**1 month**		**3 months**	**6 months** ^**∗**^	**1 year**	**2 years** ^**#**^	**3 years**	**4 years**

Total bilirubin Reference range: 0.2–1.2 mg/dL	0.5	0.3	0.3	0.6	0.3	0.7	0.6	0.5
Direct bilirubin Reference range: 0.0–0.5 mg/dL	< 0.2	< 0.2	< 0.2	0.3	< 0.2	0.3	0.2	0.2
Gamma glutamyl transferase Reference range: 12–64 U/L	—	—	49	341	126	112	53	48
White blood cell count Reference range: 4.2–9.1 K/mcL	4.67	1.71	5.08	4.35	5.21	7.97	9.76	14.89
Eosinophil percent Reference range: 0.8%–7%	12	3.5	4.1	11	4	2.1	1.7	0.8
Immunoglobulin G Reference range: 540–1822 mg/dL	724		766	1454	724	1445	1005	1153
Immunoglobulin M Reference range: 22–240 mg/dL	29		41	51	26	78	178	219
Sirolimus level Reference range: 4–20 ng/mL			10	< 2	6.6			
Tacrolimus level Reference range: 5–20 ng/mL							12	5.1
Anti-nuclear antibody Reference range: negative				Negative		1.1		
Anti-smooth muscle antibody Reference range: negative				Negative		Negative		
Antimitochondrial antibody Reference range: negative				Negative		Negative		
HEV RNA Reference range: none detected				None detected		None detected		

*Note:* U/L, units per liter; mg/dL, milligrams per deciliter; K/mcL, thousands of cells per microliter of blood; ng/mL, nanogram per milliliter.

Abbreviation: HCT, hematopoietic cell transplantation.

^∗^Time of 1^st^ biopsy diagnosis of hepatic GVHD.

^#^Time of 2^nd^ biopsy diagnosis of autoimmune-like hepatitis.

## Data Availability

The data that support the findings of this study are available from the corresponding author upon reasonable request.

## Nomenclature

GVHD: Graft-versus-host disease
HCT: Hematopoietic cell transplant
AIH: Autoimmune-like hepatitis
PTCL: Peripheral T-cell lymphoma
